# Disease-Associated PNPLA6 Mutations Maintain Partial Functions When Analyzed in *Drosophila*

**DOI:** 10.3389/fnins.2019.01207

**Published:** 2019-11-06

**Authors:** Elizabeth R. Sunderhaus, Alexander D. Law, Doris Kretzschmar

**Affiliations:** Oregon Institute of Occupational Health Sciences, Oregon Health & Science University, Portland, OR, United States

**Keywords:** neuropathy target esterase, lipid homeostasis, spastic paraplegia/ataxia, hypogonadism, chorioretinal dystrophy, organophosphate-induced delayed neuropathy

## Abstract

Mutations in patatin-like phospholipase domain-containing protein 6 (PNPLA6) have been linked with a number of inherited diseases with clinical symptoms that include spastic paraplegia, ataxia, and chorioretinal dystrophy. PNPLA6 is an evolutionary conserved protein whose ortholog in *Drosophila* is Swiss-Cheese (SWS). Both proteins are phospholipases hydrolyzing lysophosphatidylcholine (LPC) and phosphatidylcholine (PC). Consequently, loss of SWS/PNPLA6 in flies and mice increases both lipids and leads to locomotion deficits and neurodegeneration. PNPLA6 knock-out mice are embryonic lethal, and a mutation creating an early stop codon in human PNPLA6 has only been identified in compound heterozygote patients. In contrast, disease-causing point mutations are found in homozygous patients, with some localized in the phospholipase domain while others are in a region that contains several cNMP binding sites. To investigate how different mutations affect the function of PNPLA6 in an *in vivo* model, we expressed them in the *Drosophila sws^1^* null mutant. Expressing wild-type PNPLA6 suppressed the locomotion and degenerative phenotypes in *sws*^1^ and restored lipid levels, confirming that the human protein can replace fly SWS. In contrast, none of the mutant proteins restored lipid levels, although they suppressed the behavioral and degenerative phenotypes, at least in early stages. These results show that these mutant forms of PNPLA6 retain some biological function, indicating that disruption of lipid homeostasis is only part of the pathogenic mechanism. Furthermore, our finding that mutations in the cNMP binding sites prevented the restoration of normal lipid levels supports previous evidence that cNMP regulates the phospholipase activity of PNPLA6.

## Introduction

Hereditary spastic paraplegias (HSPs) are inherited degenerative disorders that mainly affect motor neurons, leading to progressive spasticity and weakness of the lower extremities (Fink, 2013; de Souza et al., 2017; Parodi et al., 2017; Blackstone, 2018). Mutations in several genes have been shown to cause HSP, including patatin-like phospholipase domain-containing protein 6 (PNPLA6) (de Souza et al., 2017; Synofzik and Schule, 2017). The first mutations identified in PNPLA6, which is also called neuropathy target esterase (NTE), were shown to cause Spastic Paraplegia Type 39 in two affected families (Rainier et al., 2008). Subsequently, mutations in PNPLA6 have also been linked with Boucher–Neuhäuser, Gordon–Holmes, Laurence–Moon, and Oliver–McFarlane Syndrome, complex diseases with varying clinical symptoms that include hypogonadism, chorioretinal dystrophy, ataxia, spasticity, and, although less frequently, peripheral neuropathy and impaired cognitive functions (Deik et al., 2014; Synofzik et al., 2014; Topaloglu et al., 2014; Kmoch et al., 2015). PNPLA6 belongs to a family of hydrolases with at least eight members in mammals that react with different substrates such as phospholipids, triacylglycerols, and retinol esters (Kienesberger et al., 2009). PNPLA6 preferably hydrolyzes phosphatidylcholine (PC) and lysophosphatidylcholine (LPC) (Lush et al., 1998; van Tienhoven et al., 2002; Quistad et al., 2003). PC is primarily synthesized in the endoplasmic reticulum (ER), which also contains substantial amounts of PC in its membranous network (Kienesberger et al., 2009; Lagace and Ridgway, 2013). In contrast, the levels of LPC in membranes are normally quite low and it can readily leave membranes and act as a messenger by signaling through membrane receptors (van Meer et al., 2008). Elevated levels of LPC, as described in a variety of diseases, may therefore have pathogenic effects by disrupting signaling pathways (Wang and Dennis, 1999; Fuchs and Schiller, 2009; Drzazga et al., 2014; Velasco et al., 2017).

Mutations in proteins that affect lipid homeostasis have been identified as the cause of over a hundred human diseases, many of them affecting the central and peripheral nervous system, confirming the importance of lipid homeostasis in neuronal health (Garcia-Cazorla et al., 2015). However, in most cases, only a few patients have been described for each disease, and the underlying mechanisms for how these mutations lead to the clinical symptoms are still mostly unknown. In the case of PNPLA6, several animal models have been used to analyze phenotypes caused by the loss of this protein. In mice, the complete loss of PNPLA6 leads to lethality during embryogenesis around day 9 postcoitum (Moser et al., 2004). Mutant embryos show growth retardation due to failed placental development and impaired vasculogenesis in the yolk sac. Brain-specific knock-out mice initially develop normally, but they exhibit defects in motor coordination and neuronal degeneration when aged (Akassoglou et al., 2004). Knocking out PNPLA6 specifically in glia resulted in incomplete ensheathment of Remak fibers by non-myelinating Schwann cells in adult sciatic nerves (McFerrin et al., 2017). In agreement with these phenotypes, PNPLA6 is expressed in Schwann cells in the peripheral nervous system starting around post-natal day 5 (McFerrin et al., 2017). PNPLA6 is also expressed in the nervous system during development, first being detectable in spinal ganglia around day 13 postcoitum (Moser et al., 2000). Postnatally, PNPLA6 is expressed in all or most neurons; however, its distribution becomes more restricted during aging, with strong expression levels persisting in large neurons within the cortex, olfactory bulb, thalamus, hypothalamus, pons, and medulla oblongata (Glynn et al., 1998; Moser et al., 2000).

PNPLA6 is an evolutionary conserved protein, and its *Drosophila* ortholog is encoded by the *swiss-cheese* (*sws*) gene (Lush et al., 1998; Moser et al., 2000). Similar to PNPLA6, SWS is expressed in most neurons when the flies are young, but as PNPLA6, it becomes primarily restricted to large neurons with age (Muhlig-Versen et al., 2005). In addition, SWS is expressed by ensheathing glia that form processes around axons, analogous to the expression of PNPLA6 in Schwann cells (Dutta et al., 2016). SWS is also enriched in the ER, and its loss causes structural alterations in the ER (Muhlig-Versen et al., 2005). Mutant *sws* flies show locomotion deficits and neuronal degeneration, both of which progress with age (Kretzschmar et al., 1997; Dutta et al., 2016). Furthermore, SWS exhibits phospholipase activity, and *sws* mutants have increased levels of PC and LPC (Muhlig-Versen et al., 2005; Kmoch et al., 2015). In addition to the phospholipase domain, both *Drosophila* SWS and mammalian PNPLA6 contain domains that are predicted to bind cAMP or cGMP and a domain that binds to catalytic subunits of protein kinase A (PKA). For SWS, it has been shown that the latter is indeed required for binding to the C3 catalytic domain of PKA (PKA-C3), whereby SWS inhibits the activity of PKA-C3 (Bettencourt da Cruz et al., 2008). These similarities indicate that SWS and PNPLA6 are both structurally and functionally conserved, which we confirmed by demonstrating that expression of either mouse or human PNPLA6 restored SWS function in *Drosophila* (Muhlig-Versen et al., 2005; Topaloglu et al., 2014). We therefore have now used the fly SWS model to investigate the consequences that different disease-associated PNPLA6 mutations have on the functions of this protein.

## Materials and Methods

### *Drosophila* Stocks

The *sws*^1^ allele has been described in Kretzschmar et al. (1997). *Appl*-GAL4 was kindly provided by L. Torroja (Universidad Autonoma de Madrid, Spain) and *loco*-GAL4 by C. Klämbt (Universitat Muenster, Muenster, Germany). The sws^JF03428^ RNAi line was obtained from the Bloomington Stock Center. To obtain the transgenic lines expressing human PNPLA6, a human cDNA encoding transcript variant 2 (NM_006702.2) was used and the mutations introduced by site-directed mutagenesis using the Quikchange Lightning site-directed mutagenesis kit according to the instructions of the manufacturer (Stratagene, La Jolla, CA, United States). The location of the substituted amino acids is referring to the PNPLA6 protein annotated as Q8IY17, as listed in UniProtKB. The PNPLA6 cDNAs were cloned into pUASTattB and integrated into the genome at the 68E attP site using the PhiC31 integrase-mediated transgenesis system (Bischof et al., 2007) and the BestGene transformation service. The lines expressing wild-type PNPLA6 and PNPLA6^D376GfsX18^ are described in Topaloglu et al. (2014). Flies were maintained on standard fly food under a 12:12 h light:dark cycle. Stocks were maintained at 18°C while crosses and aging flies were maintained at 25°C.

### Western Blots

Adult fly heads were dissected on an ice-cold plate, homogenized in RIPA lysis buffer [150 mM NaCl, 1% DOC, 1% SDS, 50 mM Tris, 5 mM ethylenediaminetetraacetic acid (EDTA), 5 mM ethylene glycol-bis(β-aminoethyl ether)-N,N,N′,N′-tetraacetic acid (EGTA), 1% triton X-100, and protease inhibitors (Cell Signaling Technology 5872S)] at 5 μl per head, and centrifuged at 25,000 × *g* for 20 min at 4°C. The supernatant was kept, mixed with NuPAGE LDS Sample Buffer (ThermoFisher B0008) and beta-mercaptoethanol to a final concentration of 1.25× and 2.5%, respectively, and denatured at 85°C for 10 min. Samples containing the equivalent of one to two heads were electrophoresed through 8% bis–tris gels (ThermoFisher NW00082) and transferred onto PVDF membranes (ThermoFisher T2234). To detect PNPLA6, we used a biotinylated secondary antibody and Streptavidin-conjugated HRP (Vector Labs PK-6200) following the manufacturer protocol with the exception that all antibodies were diluted in 1× casein blocking buffer (Sigma C7594) and all washing steps were carried out with 1× TBST (Tris-buffered saline + 0.05% TWEEN-20). To detect GAPDH, the primary antibody was diluted in 1× casein and subsequent washing and secondary antibody steps were carried out in 1× TBST. Enhanced chemiluminescent substrate (Michigan Diagnostics FWPD02) was used to visualize bands. The antisera/antibodies were used at the following dilution: rabbit anti-PNPLA6/NTE (Chen et al., 2007) (1:100; provided by Y.-J. Wu, Chinese Academy of Sciences), mouse anti-GAPDH G-9 (1:1000; Santa Cruz sc-365062), Biotinylated Goat Anti-Rabbit (1:200; Vector Labs BA-1000), and goat anti-mouse peroxidase conjugate (1:10,000; Jackson ImmunoResearch 115-035-166). For the quantification of protein levels, the intensity of the PNPLA6 bands was measured and normalized to GAPDH using Fiji (Schindelin et al., 2012). The levels of the mutant PNPLA6 proteins were then compared to the mean level of wild-type PNPLA6. Statistical analysis was done using measurements from five independent Western blots and GraphPad Prism with one-way ANOVA and a Dunnett’s *post hoc* test.

### Fast Phototaxis

Fast phototaxis assays were conducted in the dark using the countercurrent apparatus described by Benzer (1967) and a single light source. A detailed description of the experimental conditions can be found in Strauss and Heisenberg (1993). Flies were starved overnight, but had access to water and were tested the following morning. Five consecutive tests were performed in each experiment with a time allowance of 6 s to make a transition toward the light and into the next vial. Flies were obtained from four to five independent crosses from which the progeny with the correct genotype was collected every day and pooled into one vial for aging. Vials were exchanged with fresh ones every 4–5 days. Flies were then tested in groups of five to nine flies and a value determined for each fly based on which vial it reached; flies that reached the last of the six vials were scored as 100% performance, reaching the fifth vial 80%, the fourth vial 60%, the third vial 40%, the second vial 20%, and flies not leaving the first vial were scored at 0%. Progeny collected from at least four different days were tested. Statistical analysis was done using GraphPad Prism and one-way ANOVA with Dunnett’s *post hoc* tests.

### Tissue Sections and Measurement of Vacuolar Pathology

Flies were obtained and aged as described for the phototaxis experiments. Paraffin sections for light microscopy were prepared and analyzed for vacuole formation as described in Botella et al. (2003) and Sunderhaus and Kretzschmar (2016). Briefly, whole flies were fixed in Carnoy’s solution and dehydrated in an ethanol series followed by incubation in methyl benzoate before embedding in paraffin. Sections were cut at 7 μm and analyzed with a Zeiss Axioskope 2 microscope using the auto-fluorescence caused by the dispersed eye pigment. To quantify the vacuolization, we photographed sections at the level of the great commissure and numbered the pictures for a double-blind analysis. The area of the vacuoles in the deutocerebral neuropil was then calculated in ImageJ as total pixel number, converted into μm^2^, and the genotype determined. Statistical analysis was done using GraphPad Prism and one-way ANOVA with Dunnett’s *post hoc* tests.

### Lipid Analysis

Forty *Drosophila* heads were collected and pulverized in cold methyl *tert*-butyl ether:methanol:water using a ceramic bead blaster, centrifuged, and the top layer was collected for UPLC-HDMS and UPLC-SWATH analyses. Five microliters of Lipidomix^TM^ was added as an internal standard to each sample. Samples were analyzed in duplicates in positive and negative ion modes. Acquired data were searched by Peakview’s^TM^ XIC Manager and LPC, PC and PE XIC (extracted ion chromatograms) lists were searched based on formula, accurate mass, isotope ratio, and msms fragmentation. Lipid measurements were conducted at the Mass Spectrometry Facility of the Oregon State University. LPC, PC, and PE were determined and compared to one of the two *sws*^1^ samples added to each measurement. Two independently prepared sets, containing extracts from all genotypes, were used and two independent measurements obtained from each set. PC and LPC levels were normalized to PE, which we previously showed to be unchanged in *sws*^1^ (Muhlig-Versen et al., 2005). Statistical analysis was done using GraphPad Prism and one-way ANOVA with Dunnett’s *post hoc* tests or Student’s *t*-tests.

## Results and Discussion

**FIGURE 1:**
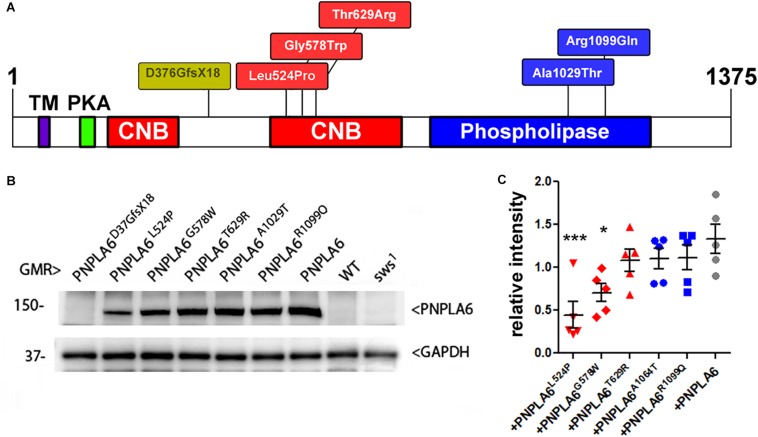
**(A)** Schematic of PNPLA6 and location of the generated mutations. TM, transmembrane domain; CNB, cyclic nucleotide binding sites; PKA, domain interacting with the PKA catalytic subunit. **(B)** Western blot showing expression levels of the PNPLA6 proteins. Anti-GAPDH was used for a loading control. **(C)** A quantification from five Western blots shows a significant reduction in protein levels in PNPLA6^*L*524*P*^ and PNPLA6^G578W^. Each symbol represents the value from one blot with mean and SEM indicated. ^∗^*p* < 0.05, ^∗∗∗^*p* < 0.001 compared to the mean levels of wild-type PNPLA6.

As mentioned above, SWS and PNPLA6 contain a phospholipase domain, plus a domain similar to regulatory PKA subunits that includes three cNMP binding sites, whereby the second and the third are located next to each other. Disease-associated mutations have been identified in both domains (at this time 23 pathogenic mutations are listed at https://varsome.com/gene/PNPLA6); however, there is no clear correlation between clinical features and location of the mutations. To determine the functional consequences of these mutations, we created transgenic lines expressing different PNPLA6 variants and tested their ability to restore SWS function in the *sws*^1^ mutant line. The *sws*^1^ mutation is caused by a stop codon at the position of serine^375^, shortening the protein to about a quarter of its normal length, lacking two of its three cNMP binding sites and the entire phospholipase domain (Kretzschmar et al., 1997). Furthermore, this shortened protein could not be detected by Western blots or immunohistochemistry, suggesting that *sws*^1^ is a null mutation (Dutta et al., 2016). We previously generated flies that express either wild-type human PNPLA6 or a mutant form (PNPLA6^D376GfsX18^) and whereas wild-type PNPLA6 did rescue the neurodegenerative phenotype of the *sws*^1^ mutant, PNPLA6^D376GfsX18^ did not (Topaloglu et al., 2014). PNPLA6^D376GfsX18^ also shortens the protein to about a quarter of its length (Topaloglu et al., 2014), deleting the entire phospholipase domain and two of the three cyclic nucleotide binding domains (Figure 1A), providing a “negative control” for our experiments. The PNPLA6^D376GfsX18^ mutation is only found in compound heterozygous patients together with the pathogenic PNPLA6^R1099C^ mutation, which also failed to rescue the degenerative phenotype of *sws*^1^ (Topaloglu et al., 2014). In addition to these two constructs, we subsequently generated five new lines carrying different PNPLA6 mutations identified in patients. All of these constructs were placed under control of the Upstream Activation Sequence (UAS) that can be activated by the yeast GAL4 transcription factor (Brand and Perrimon, 1993). Three of these new constructs contained mutations in the cNMP binding sites: PNPLA6^L524P^, PNPLA6^G578W^, and PNPLA6^T629R^, while PNPLA6^A1029T^ and PNPLA6^R1099Q^ carried mutations in the phospholipase domain (Figure 1A). PNPLA6^L524P^ and PNPLA6^R1099Q^ were identified in patients diagnosed with Oliver McFarlane Syndrome, and while they showed chorioretinopathy and hypothyroidism, they did not show signs of hypogonadism or ataxia (Hufnagel et al., 2015; Kmoch et al., 2015). PNPLA6^G578W^ is a mutation causing Boucher-Neuhäuser Syndrome, and the patients expressing this mutation exhibited cerebellar atrophy, ataxia, hypogonadism, and chorioretinal dystrophy (Synofzik et al., 2014). Patients with the PNPLA6^A1029T^ and PNPLA6^T629R^ mutations exhibit hypogonadism with absent or delayed pubertal development, characteristic for Gordon–Holmes syndrome (Kotan et al., 2016). Individuals with the PNPLA6^T629R^ mutation (PNPLA6^T581R^ in the original paper due to referring to another protein isoform) showed digenic inheritance, being heterozygote for this PNPLA6 mutation and a mutation in the steroid receptor RNA activator 1 (Kotan et al., 2016). We chose these mutations to represent different syndromes as well as to include mutations in the two identified functional domains of PNPLA6. To obtain similar expression levels in our different transgenic lines, all of the PNPLA6 constructs were inserted into the same chromosomal site using the PhiC31 integrase-mediated transgenesis system (Bischof et al., 2007). To confirm this, we performed Western blots (Figure 1B) with flies expressing the PNPLA6 constructs with GMR-GAL4 (a relatively strong promoter). As expected PNPLA6^T629R^, PNPLA6^A1029T^, and PNPLA6^R1099Q^ were expressed at similar levels as wild-type PNPLA6 (Figure 1C). However, the levels of PNPLA6^L524P^ and PNPLA6^G578W^ were significantly reduced, suggesting that the mutations affect the stability of the protein. Both mutations are localized within the cyclic nucleotide binding region while the two mutations in the phospholipase domain have no significant effect on protein levels. This indicates that cyclic nucleotide binding may play a role in stabilizing the protein. However, it should be noted that the T629R mutation, which also localizes to the cyclic nucleotide binding domain, did not reduce protein levels but it is not known yet how the different mutations affect cyclic nucleotide binding.

### Mutant PNPLA6 Proteins Are Partially Functional

**FIGURE 2:**
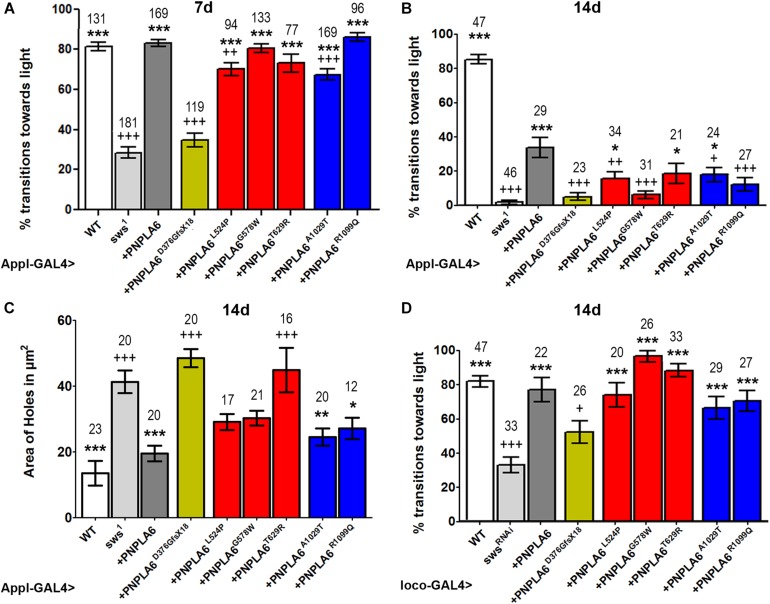
Effects of PNPLA6 expression on *sws*^1^-associated phenotypes. **(A)** Fast phototaxis assays of 7-day-old flies expressing the PNPLA6 proteins pan-neuronally with *Appl*-GAL4. Wild-type PNPLA6 and all point mutations rescue the reduced performance of *sws*^1^ while the shortened PNPLA6^D376GfsX18^ protein does not. **(B)** When the fast phototaxis assays were performed with 14-day-old flies, wild-type PNPLA6, PNPLA6^*L*524*P*^, PNPLA6^T629R^, and PNPLA6^R1099Q^ partially improved the performance. With the exception of PNPLA6^T629R^, all mutant constructs were significantly less efficient than wild-type PNPLA6 at this age. **(C)** Measuring the area of vacuoles in the deutocerebral neuropil of 14-day-old *sws*^1^ flies expressing wild-type PNPLA6 with *Appl*-GAL4 showed a suppression of the degeneration. Two of the point mutations in the phospholipase domain also suppressed the degeneration observed in *sws*^1^. **(D)** Fast phototaxis assays of 14-day-old flies co-expressing the PNPLA6 proteins and the sws^RNAi^ construct in all glia using *loco*-GAL4. All PNPLA6 construct besides PNPLA6^D376GfsX18^ suppressed the locomotion deficits of the *sws* knockdown flies. Means and SEMs are indicated. The number of analyzed individuals is given above each bar. For the phototaxis experiments in panels **A**, **B**, and **D**, the flies were tested in groups of five to nine flies. ^∗^*p* < 0.05, ^∗∗^*p* < 0.01, ^∗∗∗^*p* < 0.001 compared to *sws*^1^ **(A–C)**, the sws^RNAi^ **(D)**. ^+^*p* < 0.05, ^++^*p* < 0.01, ^+++^*p* < 0.001 compared to wild-type PNPLA6. One-way ANOVA with Dunnett’s *post hoc* test was used for statistical analyses.

To assess the impact of each mutation on the *in vivo* function of PNPLA6, we expressed them pan-neuronally in *sws*^1^ mutants via *Appl*-GAL4. As shown in Figure 2A, *sws*^1^ flies show a severe reduction in locomotion in the fast phototaxis assay by as early as 7 day old. Expressing wild-type PNPLA6 restored locomotory behavior to wild-type levels, demonstrating that the human protein can replace fly SWS. As expected, the PNPLA6^D376GfsX18^ mutant, which we previously showed does not rescue degenerative phenotypes in *sws*^1^ flies, failed to rescue the locomotion phenotype. In contrast, all of the other PNPLA6-mutant forms did improve locomotion (significances to *sws*^1^ indicated by ^∗^). Whereas *sws*^1^ flies expressing PNPLA6^G578W^, PNPLA6^T629R^, and PNPLA6^R1099Q^ were not significantly different from flies expressing wild-type PNPLA6, flies expressing either PNPLA6^L524P^ or PNPLA6^A1029T^ only partially restored normal locomotion (significances to wild-type PNPLA6 indicated by +). Due to the locomotor deficits in *sws*^1^ flies becoming progressively worse with age, 14-day-old *sws*^1^ flies barely move (Figure 2B). Again, this deficit was significantly improved by the induction of wild-type PNPLA6 with *Appl*-GAL4, although it did not fully restore normal locomotor behavior. This partial effect is consistent with our previous results showing that the loss of SWS in glia also causes locomotor deficits that cannot be rescued by expression of SWS in neurons (Muhlig-Versen et al., 2005; Dutta et al., 2016). PNPLA6^L524P^, PNPLA6^T629R^, and PNPLA6^A1029T^ also still partially rescued although not as efficiently as wild-type PNPLA6. With the exception of PNPLA6^T629R^, all mutant lines were significantly worse than wild-type PNPLA6, suggesting that even constructs like PNPLA6^G578W^ and PNPLA6^R1099Q^ that could prevent deleterious effects on the locomotion as efficiently as wild-type PNPLA6 in young flies cannot significantly delay the progression.

Next, we tested whether expressing the PNPLA6 constructs in neurons could ameliorate the neurodegeneration observed in *sws*^1^. For this analysis, we measured the area of vacuoles that form in 14-day-old *sws*^1^ flies (Figure 2C). As expected, expressing wild-type PNPLA6 prevented neurodegeneration, as did both PNPLA6 proteins with mutations in the phospholipase domain. In contrast, none of the constructs with mutations in the cNMP-binding region had a significant beneficial effect. Although the suppression was not significant compared to *sws*^1^, PNPLA6^*L*524*P*^ and PNPLA6^G578W^ did show some improvement and they were not significantly different from wild-type PNPLA6.

As mentioned above, SWS also has a cell-autonomous function in glia, and knocking down SWS specifically in glia results in locomotor deficits (Dutta et al., 2016). We therefore tested how each of our PNPL6 mutations affected glial function in SWS knockdown flies by expressing them in all glia using *loco*-GAL4. Testing 14-day-old flies in the fast phototaxis assay revealed that all PNPLA6 forms could suppress the locomotion defects seen in the knock-down flies, with the exception of PNPLA6^D376GfsX18^ (Figure 2D). Together our results show that human PNPLA6 can functionally replace SWS in neurons and glia, confirming previous results with either human or mouse PNPLA6 (Muhlig-Versen et al., 2005; Topaloglu et al., 2014). They also confirm that the PNPLA6^D376GfsX18^ mutation, which results in a shortened protein that has lost the phospholipase domain as well as two of the cAMP binding sites, is not functional. As mentioned above, this mutation is only found in compound heterozygous patients (Topaloglu et al., 2014) and together with our findings that the loss of PNPLA6 in mice causes lethality during embryonic development, these results support the conclusion that the complete loss of functional PNPLA6 in humans is lethal. In contrast, none of the point mutations induces a complete loss of function. Although all of these mutations do impair aspects of PNPLA6 function (see below), they are still capable of partially rescuing the degenerative phenotypes and locomotion deficits observed in the *sws*^1^ null mutant. Furthermore, we did not detect significant differences in the rescue ability of mutations associated with a specific disease, suggesting that the different phenotypic presentation may be due to genetic background effects or other individual differences rather than different effects on PNPLA6 function. It should also be noted that a specific syndrome can be caused by mutations in either the phospholipase domain or the cyclic nucleotide binding domain; like the L524P and R1099Q mutations which are both described to cause Oliver–McFarlane syndrome, or the T629R and A1029T mutations associated with Gordon–Holmes syndrome, also supporting that these syndromes are not due to effects on specific PNPLA6 functions.

### Mutations in Both Domains Interfere With the Phospholipase Function

**FIGURE 3:**
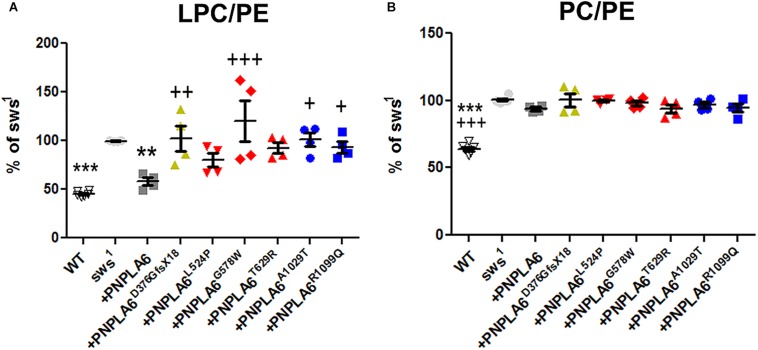
**(A)** Pan-neuronal expression of wild-type PNPLA6 reduces the LPC/PE ratio in *sws*^1^ but none of the mutations does. **(B)** None of the PNPLA6 proteins significantly reduces the PC/PE ratio in *sws*^1^. Mean from four measurements and SEM shown. Flies were 3–5 days old. Each symbol represent the value form one measurement. ^∗∗^*p* < 0.01, ^∗∗∗^*p* < 0.001 compared to *sws*^1^. ^+^*p* < 0.05, ^++^*p* < 0.01, ^+++^*p* < 0.001 compared to wild-type PNPLA6. One-way ANOVA with Dunnett’s *post hoc* test was used for statistical analyses.

Loss of PNPLA6 or SWS results in a rise in PC and LPC levels in flies and mice (Muhlig-Versen et al., 2005; Read et al., 2009; Kmoch et al., 2015), whereby LPC has been suggested to be the preferred substrate of SWS/NTE (van Tienhoven et al., 2002; Quistad et al., 2003). To determine how the different PNPLA6 mutations affect its phospholipase function, we measured LPC and PC levels in *sws*^1^ flies expressing these constructs pan-neuronally with *Appl*-GAL4. PC and LPC levels were normalized to phosphatidylethanolamine (PE), which we previously showed not to be altered in *sws*^1^ (Muhlig-Versen et al., 2005). Consistent with our previous results, we detected an increase in LPC and PC levels in 3–5-day-old *sws*^1^ flies (Figures 3A,B). Expressing wild-type PNPLA6 reduced LPC levels in *sws*^1^ flies, but the PC levels were not significantly decreased compared to *sws*^1^ when using multiple comparison tests. However in a direct comparison, *sws*^1^ flies expressing PNPLA6 did show a significant reduction (*P* = 0.002, Student’s *t*-test). In contrast, none of the mutant constructs had an effect on PC levels in *sws*^1^ flies either in direct or in multiple comparison tests (Figure 3B), nor did they improve LPC levels. Moreover, PNPLA6^D376GfsX18^, PNPLA6^G578W^, PNPLA6^A1029T^, and PNPLA6^R1099Q^ were significantly worse than PNPLA6 (Figure 3A). This shows that all mutations interfere with the phospholipase activity of PNPLA6.

Although none of the mutants could suppress the phospholipid defects, all the point mutations reduced the locomotion deficits at 7th day and the two mutations in the phospholipase domain also ameliorated neuronal degeneration when tested at 14 days. These results suggest that changes in phospholipid levels are not the only factor in the pathogenicity of PNPLA6 mutant forms, and that the mutant constructs still can fulfill other PNPLA6-dependent functions. That effects on the phospholipase activity are not sufficient to cause disease has also been suggested by measuring PNPLA6 activity in human fibroblasts. While some carriers of mutations that cause spastic paraplegia revealed even less PNPLA6 activity than the patients, they were nevertheless symptom-free (Hein et al., 2010). In addition, we found that the constructs containing mutations in the cNMP-binding sites (but with an intact phospholipase domain) were equally deficient in reducing LPC or PC, supporting previous findings that cNMP can regulate the phospholipase activity of PNPLA6 (Chen et al., 2010). Lastly, whereas expressing wild-type PNPLA6 could reduce the levels of LPC to near-control levels it had a much weaker effect on PC. Nevertheless, PNPLA6 did suppress the locomotion and degenerative phenotypes of *sws*^1^. We previously obtained similar results by activating ER stress responses in *sws*^1^, which also reduced LPC levels but not PC levels, as well as suppressing behavioral and degenerative phenotypes (Sunderhaus et al., 2019). Together, these results suggest that the rise in LPC is more toxic than the increased PC. Whereas PC is a major component of all cell membranes, the concentration of LPC is normally quite low (Kienesberger et al., 2009; Lagace and Ridgway, 2013). Moreover, increased levels of LPC have been linked with a variety of diseases, including demyelination and neuropathic pain (Wang and Dennis, 1999; Fuchs and Schiller, 2009; Drzazga et al., 2014; Velasco et al., 2017). Although we did not detect significant changes in myelin in a glial-specific knock-out of PNPLA6 in mice, it did cause an incomplete ensheathment of Remak bundles by non-myelinating Schwann cells in the sciatic nerve (McFerrin et al., 2017). However, lipid composition also regulates vesicle fusion and therefore synaptic transmission (Lauwers et al., 2016). Furthermore, LPC can promote apoptosis by increasing the pro-apoptotic proteins Bax and cleaved caspase-3 (Lin and Ye, 2003; Kakisaka et al., 2012; Wang et al., 2016; Chang et al., 2017). Therefore, reducing only LPC levels may be sufficient to suppress the behavioral and degenerative phenotype of *sws*^1^.

## Data Availability Statement

The datasets generated for this study are available on request to the corresponding author.

## Author Contributions

ES performed the behavioral and anatomical experiments, and provided the samples for the lipid measurements. AL performed the Western blot experiments. ES and DK designed and analyzed the experiments. DK wrote the manuscript.

## Conflict of Interest

The authors declare that the research was conducted in the absence of any commercial or financial relationships that could be construed as a potential conflict of interest.
